# Object orientated automated image analysis: quantitative and qualitative estimation of inflammation in mouse lung

**DOI:** 10.1186/1746-1596-3-S1-S16

**Published:** 2008-07-15

**Authors:** Coralie Apfeldorfer, Kristina Ulrich, Gareth Jones, David Goodwin, Susie Collins, Emanuel Schenck, Virgile Richard

**Affiliations:** 1 Pfizer Ltd., DSRD, Ramsgate Road, Sandwich, Kent CT13 9NJ, UK

## Abstract

Historically, histopathology evaluation is performed by a pathologist generating a qualitative assessment on thin tissue sections on glass slides. In the past decade, there has been a growing interest for tools able to reduce human subjectivity and improve workload. Whole slide scanning technology combined with object orientated image analysis can offer the capacity of generating fast and reliable results. In the present study, we combined the use of these emerging technologies to characterise a mouse model for chronic asthma. We monitored the inflammatory changes over five weeks by measuring the number of neutrophils and eosinophils present in the tissue, as well as, the bronchiolar associated lymphoid tissue (BALT) area on whole lungs sections. We showed that inflammation assessment could be automated efficiently and reliably. In comparison to human evaluation performed on the same set of sections, computer generated data was more descriptive and fully quantitative. Moreover optimisation of our detection parameters allowed us to be to more sensitive and to generate data in a larger dynamic range to traditional experimental evaluation, such as bronchiolar lavage (BAL) inflammatory cell counts obtained by flow cytometry. We also took advantage of the fact that we could increase the number of samples to be analysed within a day. Such optimisation allowed us to determine the best study design and experimental conditions in order to increase statistical significance between groups. In conclusion, we showed that combination of whole slide digital scanning and image analysis could be fully automated and deliver more descriptive and biologically relevant data over traditional methods evaluating histopathological pulmonary changes observed in this mouse model of chronic asthma.

## Introduction

Obtaining quantitative data from histological sections represents a formidable challenge. While an investigator might have the patience to count objects in a few given fields, the task is tedious and may even result in inconsistent classification across investigators. Not surprisingly, much effort has been devoted to automating the classification and quantification of histological structures in various disease models [1-7]. We will show here that Definiens eCognition tools are able to generate accurate and powerful algorithms allowing us to process morphometric data faster and more reliably than traditional slide scoring in a mouse model of chronic asthma.

## Materials and methods

### Animal treatment

Female BALB/c mice (6–8 weeks, 16–22 g, Charles River, UK) were housed under specific pathogen-free conditions. Mice were exposed to purified HDM extract (Dermatophagoides pteronyssinus; Greer Laboratories, Lenoir, NC) intranasally (1, 5, 10 and 25 μg in 10 μl of saline) while under transient isoflurane anaesthesia. Control animals received 10 μl saline intranasally. Exposures were carried out for 5 days/week for up to 5 weeks.

### Section preparation

After BAL had been performed, the lungs were removed from the thoracic cavity and expanded with 10% formalin (Sigma, UK) through the tracheal cannula. The lung was immersed in 10% formalin and fixed for 24 hours at room temperature, prior to being placed dorsal surface down into a cassette and processed into paraffin wax (Tissue-Tek VIP, ThermoFisher Scientific). Sectioning was carried out at 4.5 μm. Three sections (or more) which were at least 10 μm apart, were evaluated from each animal using the following protocols: 1. Tissue eosinophil IHC using a primary antibody to the Major Basic Protein (1/100 dilution, goat anti-EMBP(S-16), Santa Cruz Biotechnology, Heidelberg, Germany) and biotinylated donkey anti-goat IgG (1/500 dilution, Jackson ImmunoResearch, Stratech, UK). The complex was visualised with hydrogen peroxide substrate and DAB chromogen (Ventana, US). 2. Tissue neutrophils IHC using a primary antibody to the Myeloperoxidase (1/50 dilution, Rabbit anti Myeloperoxidase from Abcam ab9535) and biotinylated donkey anti-goat IgG (1/200 dilution, Jackson ImmunoResearch, Stratech, UK). The complex was visualised with hydrogen peroxide substrate and DAB chromogen (Ventana, US). 3. Periodic Acid Schiff (PAS) staining for carbohydrate macromolecules in bronchiolar mucus using a Leica ST5020 autostainer (Leica Microsystems, UK). The slides were cleaned and digitalised with the Nanozoomer Digital Pathology System (NDP, Hamamatsu, Welwyn Garden City, UK) within 2 days of staining at 20 × magnification.

### Ruleset design

The algorithms used for image analysis were developed under Definiens 6.0. First, tissue sections were recognised at 1× virtual magnification (according to objects brightness), allowing us to analyse separately sections located on a single glass slide. A specific ruleset was developed for each biomarker, allowing us to detect and measure several parameters for each staining. Numerous internal measurements were performed along the different categories of tissue search to obtain auto-adjusting thresholds. Almost no size or shape criteria were used, favouring context information.

### Ruleset validation and statistical analysis

The ruleset was validated by comparing results generated by Definiens 6.0 on a small subset of images (15) with manual or semi-automated evaluation (Image Pro Plus). Data were graphed and analyzed by Microsoft Excel and are expressed as mean ± SEM. Inflammatory index was calculated as the cubic root of the relative number of neutrophils (to week one saline group) multiplied by the relative number of eosinophils (to week one saline group) multiplied by the relative area of bronchiolar associated lymphoid tissue (to week one saline group).

## Results

### Inflammatory state evaluation is more descriptive and accurate than pathologist assessment

**Figure 1:**
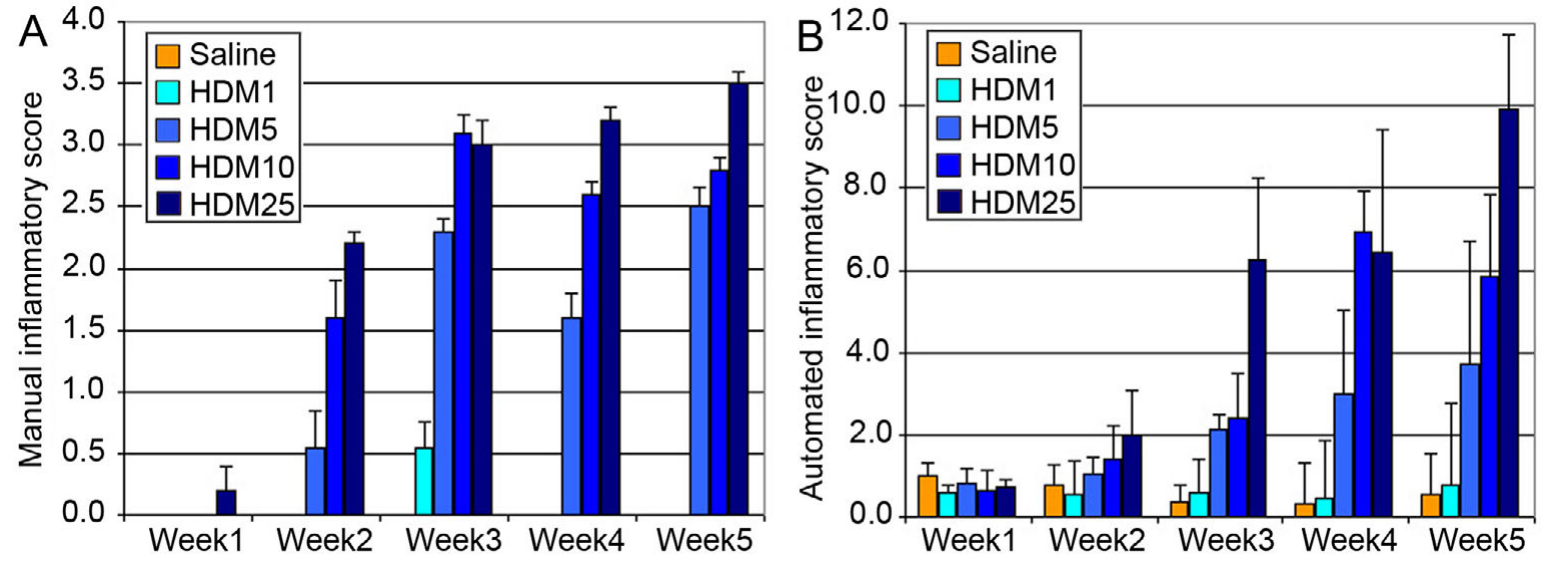
Comparison between manual and computerised assessment of pulmonary inflammation in mouse receiving House Dust Mite (HDM) extracts over 5 weeks. A. Manual assessment of pulmonary inflammation in mouse performed by a pathologist. B. Automated assessment of pulmonary inflammation done by combining 3 signs of inflammation: total number of neutrophils detected, total number of eosinophils detected and total area covered by inflammatory cells.

We first assessed manually the pulmonary inflammatory changes in mice receiving house dust mite extracts using standard histopathological criteria. This scoring was done on scale from 0 to 4 evaluating the different components associated with inflammation, where 0 is not inflamed tissue and 4 very inflamed tissue (figure 1A). We then tried to reproduce the cognitive assessment performed by the pathologist to attribute this score, by measuring the number of eosinophils, the number of neutrophils and the total area covered by bronchiolar associated lymphoid tissue. Those three parameters where combined as described in Material and methods to generate an inflammatory index (figure 1B).

Direct comparison between manual assessment and fully automated assessment of the inflammatory changes in lung sections shows a linear correlation with r2 = 0.6414. We can notice that at low HDM dosage, the pathologist often gives an inflammatory score of zero, which is not the case for the computer. In fact, directly comparing 10 μg and 25 μg dosage only, shows a better correlation with r2 = 0.8855. The best correlation is given for HDM dosage of 25 μg with r2 = 0.9654. This tends to indicate that the computer is able to detect more subtle changes within the tissue compared to human scoring and therefore reinforce the objectivity and quantitative aspect of this computer-assisted image analysis approach.

Interestingly, the automated scoring was given by the combination of 3 independent measurements, which taken individually can be more descriptive physiologically than a simple inflammatory scoring. We can determine the kinetics of neutrophils and eosinophils infiltration within the lungs, helping us to better understand the inflammatory mechanisms associated with this current model.

### Proper normalisation factor multiply study power and sensitivity

**Figure 2:**
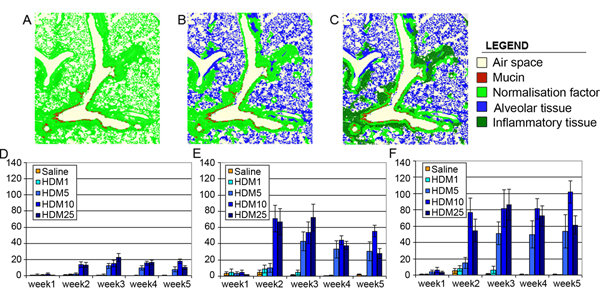
Influence of the normalisation factor on mucin secretion detection levels. A, B and C Panels show classification views of a mouse tissue section after analysis. The red surface represents the area covered by mucin (our nominator), and the light green surface represents the tissue area used for normalisation purposes (our denominator). We used at first the whole tissue section (excluding the air space) as shown in panel A, which generated the results shown in the graph D. We then excluded alveolar tissue as shown in panel B, and generated the results shown in the graph E. At last we excluded inflammatory infiltrate as shown in panel C, and generated the results shown in the graph F.

Whilst we have shown that our automated system can be biologically more relevant than simple pathologist scoring, we also tested the sensitivity and dynamic range of our system. As one could expect, surface measurement such as bronchiolar associated lymphoid tissue or mucin only make sense if they are normalised to the area of tissue effectively analysed. We have shown in Figure 2 that changing the normalising factor of mucin area measurement for example drastically improves our results.

When we measure the area of mucin relative to the tissue area (excluding the airspace) we can monitor a relative average 20 fold increase between control group and highest dosage of HDM (Figure 2D). As a large part of the tissue cannot constitutively contain any mucin we excluded alveolar tissue area from our normalisation factor. This does increase the dynamic range of our measurements, giving us an average 60 fold increase between our control group and the highest dosage of HDM (Figure 2E). Unfortunately, as we have previously shown, the HDM treatment also induces an increase in bronchiolar associated lymphoid tissue area. This introduces a bias in mucin coverage estimation, and was removed from our normalisation calculation. We finally obtain an average 80 folds increase between our control group and the highest dosage of HDM (Figure 2F).

At necropsy, a bronchiolar lavage (BAL) was performed on the lung prior to perfusion with fixative. This lavage solution was analysed by flow cytometry in order to determine the proportion of eosinophils and neutrophils in the lung fluids (data not shown). Interestingly, we obtain an excellent correlation between BAL results and image analysis (r2 = 0.8213 for neutrophils, r2 = 0.9198 for eosinophils) indicating a high level of accuracy. Nevertheless our correlation where not entirely linear, indicating a higher sensitivity in low concentration of neutrophils (about 2 folds) and a larger dynamic range in large concentrations of eosinophils.

### Number of sections has little influence on statistical power of the study

We have shown that this technique is reliable, descriptive and more sensitive than manual or flow cytometry assessment. One other advantage of automated analysis is that is can be run fairly quickly once it has been optimised. We have been able to analyse one image every 2 minutes in the case of eosinophils and neutrophils count and one image every 5 minutes in the case of mucin detection. Because of this fact, we assessed if increasing the number of serial sections per animal would further increase the statistical significance of study data, allowing us to optimize the number of animals per group. As shown on table 1, we have seen little improvement of statistically relevant detection level when increasing the number of sections compared to the influence of the number of animal per group.

**Table 1:** Relative influence of the number of sections per animal and the number of animals per group on mucin detection levels. The numbers shown here represent the fold of changes over control group required to reach statistical significance.

Mucin	Number of animals							
	3	4	5	6	7	8	9	10
	
2 sections	4.78	3.39	2.83	2.52	2.32	2.17	2.07	1.98
3 sections	4.22	3.08	2.61	2.34	2.17	2.04	1.95	1.88
4 sections	3.95	2.92	2.50	2.25	2.09	1.98	1.89	1.82
5 sections	3.79	2.83	2.43	2.20	2.05	1.94	1.85	1.79

Eosinophils	Number of animals							

	3	4	5	6	7	8	9	10
	
2 sections	8.79	5.46	4.25	3.61	3.22	2.94	2.74	2.58
3 sections	7.65	4.90	3.88	3.33	2.98	2.75	2.57	2.43
4 sections	7.11	4.63	3.69	3.19	2.87	2.65	2.48	2.35
5 sections	6.80	4.47	3.58	3.10	2.80	2.59	2.43	2.31

Neutrophils	Number of animals							

	3	4	5	6	7	8	9	10
	
2 sections	3.89	2.89	2.47	2.23	2.07	1.96	1.88	1.81
3 sections	3.69	2.77	2.38	2.16	2.02	1.91	1.83	1.77
4 sections	3.59	2.71	2.34	2.13	1.99	1.89	1.81	1.75
5 sections	3.53	2.68	2.31	2.11	1.97	1.87	1.79	1.73

This statistical analysis confirms that we are monitoring accurately the pulmonary inflammatory changes with 2 or 3 sections analysed per animal when having at least 5 animals per group. Similar statistical analysis on other tissues and biomarkers will be influenced by the complexity of the organ evaluated and the frequency and distribution of the biomarker measured. Indeed, one should expect different results in the case of more heterogeneous tissue such as brain, or cancerous lesions, for example. Nevertheless, we have demonstrated a powerful tool allowing us to make such a quality assessment.

## Discussion

As we have seen, the use of automated image analysis can give accurate readings of pulmonary inflammatory changes in mice. By using whole slide scanning instead of stereology we reduce the chance of missing a potentially interesting outlier without increasing processing time and workload in slide production. We therefore generate quantitative and objective results. We can rapidly and automatically assess pulmonary inflammation, thereby reducing human workload and bias. Moreover by combining different types of analysis, we can analyse complex structures and generate biologically descriptive data.

Taken together the data we presented are showing that these tools can help generate reliable, comprehensive and biologically descriptive data in a sensitive and time effective fashion.
